# Fusion of *piggyBac*-like transposons and herpesviruses occurs frequently in teleosts

**DOI:** 10.1186/s40851-018-0089-8

**Published:** 2018-02-21

**Authors:** Yusuke Inoue, Masahiko Kumagai, Xianbo Zhang, Tomonori Saga, Deshou Wang, Akihiko Koga, Hiroyuki Takeda

**Affiliations:** 1 Department of Biological Sciences, Graduate School of Science, The University of Tokyo, 7-3-1 Hongo, Bunkyo-ku, Tokyo, 113-0033 Japan; 2 Key Laboratory of Freshwater Fish Reproduction and Development (Ministry of Education), Key Laboratory of Aquatic Science of Chongqing, School of Life Sciences, Southwest University, Chongqing, 400715 China; 3 Primate Research Institute, Kyoto University, 41-2 Kanrin, Inuyama, Aichi 484-8506 Japan

**Keywords:** Endogenous viral elements, Herpesvirus, Transposon, *piggyBac*, Teleosts

## Abstract

**Background:**

Endogenous viral elements play important roles in eukaryotic evolution by giving rise to genetic novelties. Herpesviruses are a large family of DNA viruses, most of which do not have the ability to endogenize into host genomes. Recently, we identified a novel type of endogenous herpesvirus, which we named “*Teratorn*”, from the medaka (*Oryzias latipes*) genome, in which the herpesvirus is fused with a *piggyBac*-like DNA transposon, forming a novel mobile element. *Teratorn* is a unique herpesvirus that retains its viral genes intact and has acquired the endogenized lifestyle by hijacking the transposon system. However, it is unclear how this novel element evolved in the teleost lineage and whether fusion of two mobile elements is a general phenomenon in vertebrates.

**Results:**

Here we performed a comprehensive genomic survey searching for *Teratorn*-like viruses in publicly available genome data and found that they are widely distributed in teleosts, forming a clade within *Alloherpesviridae*. Importantly, at least half of the identified *Teratorn*-like viruses contain *piggyBac*-like transposase genes, suggesting the generality of the transposon-herpesvirus fusion in teleosts. Phylogenetic tree topologies between the *piggyBac*-like transposase gene and herpesvirus-like genes are nearly identical, supporting the idea of a long-term evolutionary relationship between them.

**Conclusion:**

We propose that *piggyBac*-like elements and *Teratorn*-like viruses have co-existed for a long time, and that fusion of the two mobile genetic elements occurred frequently in teleosts.

**Electronic supplementary material:**

The online version of this article (10.1186/s40851-018-0089-8) contains supplementary material, which is available to authorized users.

## Background

Viruses are numerically the most abundant organismal entities on earth and their interactions with host organisms range from symbiosis to infectious disease. Occasionally, viruses are integrated into the chromosomes of germline cells and become a heritable part of the host genome. These are referred to as endogenous viral elements (EVEs) [1, 2].

Herpesviruses are double-stranded DNA viruses that infect a wide variety of animals, from vertebrates to invertebrates (e.g. molluscs) [3]. They have relatively large genomes ranging from 124- to 295-kb in length [3, 4], and sometimes cause symptoms such as herpes zoster and lymphoma in mammals [5] and intestinal inflammation and epithelial necrosis in teleost fishes [3, 6, 7]. Despite their diversity, nearly all herpesvirus species reported to date establish episomal latency in the nucleus of target cells until recurrent reactivation; chromosomal integration does not occur during episomal latency [5]. Thus, herpesviruses are not usually present in the form of EVEs. The only known exceptions to this tendency are human herpesvirus 6 (HHV-6) and tarsier endogenous herpesvirus, both of which integrated into the telomeric region via homologous recombination [8–11].

Recently, we identified another type of endogenized herpesvirus, “*Teratorn*”, from the genome of the small teleost fish medaka (*Oryzias latipes*) (Inoue et al., 2017 [12]). *Teratorn* retains the capacity to transpose and is the result of a unique fusion of a functional *piggyBac*-like transposon and the whole genome of a herpesvirus (tentatively named “*Teratorn*-like virus”). Thus, the *Teratorn*-like virus is thought to have acquired the endogenized lifestyle by hijacking the transposon system. Phylogenetic analysis showed that *Teratorn*-like virus belongs to the family *Alloherpesviridae*, which preferentially infects fish and amphibians [12]. To our knowledge, *Teratorn*-like virus is the first virus to use DDE transposase (except for retrovirus-like integrase) for endogenization in eukaryotes. In addition, we previously found that fusion of the *piggyBac*-like element and herpesvirus is not restricted to medaka, but rather occurs in four other teleost fish species (yellow croaker, Nile tilapia, turquoise killifish, and ocean sunfish) [12], which led us to speculate that fusion of these two mobile elements occurred frequently in fish. Furthermore, it has recently been reported that sequences of *Teratorn*-like viruses are widely distributed in teleost genomes [13]. However, it remains unclear how general the fusion event is and how the relationship between *piggyBac*-like elements and *Teratorn*-like viruses became established; i.e., whether the fusion was a mere accident or the result of a long-term intimate relationship. In order to gain an understanding of the evolutionary relationship between these two genetic elements, we performed a comprehensive search for *Teratorn*-like viruses and *piggyBac*-like elements in vertebrates and compared their phylogeny.

## Results

### *Teratorn*–like viruses are widely distributed in teleosts

**Fig. 1:**
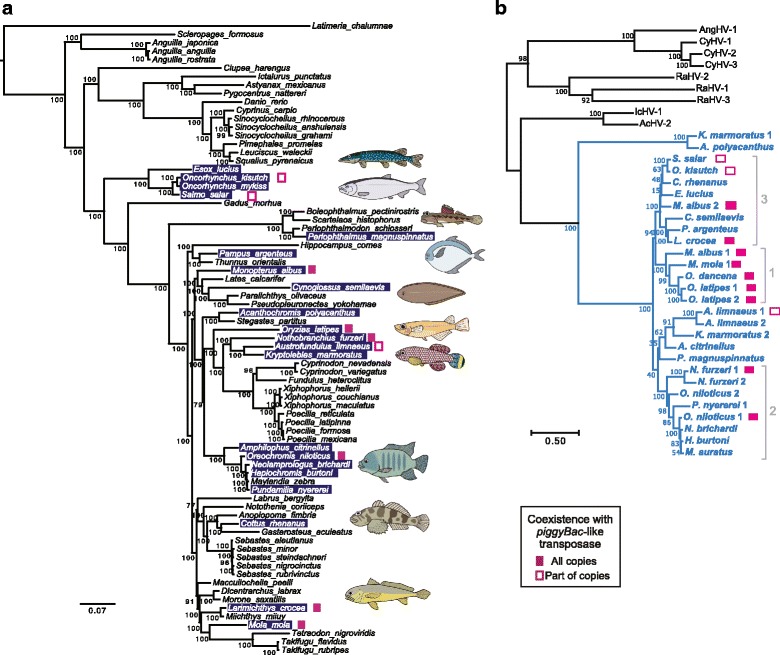
*Teratorn*-like viruses are widely distributed in teleost fish genomes (**a**) Result of a tblastn search for 13 herpesvirus core genes of medaka *Teratorn* against publicly available genome data of teleost fish species. Species that appear to contain *Teratorn*-like virus (> 8 of the 13 herpesvirus core genes; E-value < 10^− 3^) are highlighted in blue. The phylogenetic tree was constructed by Bayesian inference, based on the concatenated nucleotide sequence of 17 host genes (Betancur-R. R. et al., 2013 [39]). Species in which *Teratorn*-like viruses are adjacent to a *piggyBac*-like transposase gene are marked by magenta squares (solid, fusion for all copies; outlined, co-localization for only some of the copies). **b** Maximum-likelihood tree based on the concatenated amino acid sequences of five herpesvirus genes (major capsid protein, capsid triplex protein, DNA helicase, DNA polymerase and DNA packaging terminase) from *Teratorn*-like viruses in teleosts and exogenous alloherpesvirus species. *Teratorn*-like viruses are depicted in blue. Elements that harbor a *piggyBac*-like transposase gene are marked by magenta squares. For the designated numbers on the right (1, 2, 3), see Fig. 4c and main text. The scale bars represent the number of substitutions per site

To address the distribution of *Teratorn*-like viruses in other organisms, we performed a blast search against a publicly available vertebrate genome dataset. Tblastn search of 13 herpesvirus core genes of medaka *Teratorn* showed that *Teratorn*-like sequences are present in at least 22 of the 77 teleost fish species (E-value < 10^− 3^, more than 8 of the 13 core genes, Fig. 1a, Table 1, Additional file 1: Figure S1, Additional file 2: Table S1). For about half of these 22 species, we obtained multiple genomic loci by blast search of each herpesvirus-like gene, suggesting that *Teratorn*-like viruses are present in multiple copies in those species (see below). In addition, in some species, there are more than two subtypes of *Teratorn*-like viruses within a single species (pairwise nucleotide sequence identity < 90%, Additional file 1: Figure S1, Table 2). For each subtype, however, sequence identity between copies is high (pairwise nucleotide sequence identity > 95%).

**Table 1:** tblastn of alloherpesvirus core genes of medaka *Teratorn* against teleost genomes

Species	pol	hel	pri	mcp	tri	mem	ter	pro	37	54	56	60	64	No. of genes
*A. citrinellus*	◎	◎	◎	◎	◎	◎	◎	◎	◎	◎	◎	◎	◎	13
*A. limnaeus*	◎	◎	◎	◎	◎	◎	◎	◎	◎	◎	◎	◎	◎	13
*C. rhenanus*	◎	◎	◎	◎	◎	◎	◎	◎	◎	◎	◎	◎	◎	13
*E. lucius*	◎	◎	◎	◎	◎	◎	◎	◎	◎	◎	◎	◎	◎	13
*K. marmoratus*	◎	◎	◎	◎	◎	◎	◎	◎	◎	◎	◎	◎	◎	13
*L. crocea*	◎	◎	◎	◎	◎	◎	◎	◎	◎	◎	◎	◎	◎	13
*M. albus*	◎	◎	◎	◎	◎	◎	◎	◎	◎	◎	◎	◎	◎	13
*N. brichardi*	◎	◎	◎	◎	◎	◎	◎	◎	◎	◎	◎	◎	◎	13
*N. furzeri*	◎	◎	◎	◎	◎	◎	◎	◎	◎	◎	◎	◎	◎	13
*O. kisutch*	◎	◎	◎	◎	◎	◎	◎	◎	◎	◎	◎	◎	◎	13
*O. niloticus*	◎	◎	◎	◎	◎	◎	◎	◎	◎	◎	◎	◎	◎	13
*O. latipes*	◎	◎	◎	◎	◎	◎	◎	◎	◎	◎	◎	◎	◎	13
*P. argenteus*	◎	◎	◎	◎	◎	◎	◎	◎	◎	◎	◎	◎	◎	13
*P. magnuspinnatus*	◎	◎	◎	◎	◎	◎	◎	◎	◎	◎	◎	◎	◎	13
*P. nyererei*	◎	◎	◎	◎	◎	◎	◎	◎	◎	◎	◎	◎	◎	13
*S. salar*	◎	◎	◎	◎	◎	◎	◎	◎	◎	◎	◎	◎	◎	13
*H. burtoni*	◎	◎	◎	◎	◎	◎	◎		◎	◎	◎	◎	◎	12
*C. semilaevis*	◎		◎	◎	◎		◎	◎	◎	◎	◎	◎	◎	11
*M. auratus*	◎	◎	◎	◎	◎	◎	◎			◎	◎	◎	◎	11
*M. mola*	◎	◎		◎	◎	◎	◎	◎		◎	◎	◎		10
*O. mykiss*	◎		◎			◎	◎	◎		◎	◎	◎	◎	9
*A. polyacanthus*	◎		◎	◎			◎		◎	◎	◎	◎		8
*L. bergylta*	◎		◎		◎			◎	◎		◎			6
*M. peelii*				◎			◎		◎	◎		◎		5
*M. zebra*						◎	◎			◎	◎		◎	5
*M. saxatilis*				◎	◎	◎				◎		◎		5
*B. pectinirostris*	◎						◎			◎		◎		4
*L. fuelleborni*				◎		◎			◎		◎			4

*Abbreviations: pol* DNA polymerase, *hel* DNA helicase, *pri* primase *mcp* major capsid protein, *tri* capsid triplex protein, *mem* membrane protein, *ter* DNA packaging terminse, *pro* capsid maturation protease; 37, ORF37 of Ictalurid herpesvirus 1; 54, ORF54; 56, ORF56; 60, ORF60; 64, ORF64

**Table 2:** Characteristics of *Teratorn*-like viruses within teleost genomes

Species	Intactness of ORFs	Copy No. /haploid	Validity of Genomic integration	*piggyBac*-herpesvirus fusion	Terminal sequences	Subtypes
*S. salar*	intact	~ 8	integrated (contig-mediated link between *Teratorn*-like virus and genomic region)	Some *piggyBac* copies exist near *Teratorn*-like virus	TIRs of *piggyBac*-like element exist for a single contig (Aswad A. and Katzourakis A., 2017)	
*O. mykiss*	degraded	–	integrated (ORF degradation)	–	Unidentified (degradation of *Teratorn*-like virus sequences)	
*O. kisutch*	intact	1–5	integrated (contig-mediated link between *Teratorn*-like virus and genomic region)	Some *piggyBac* copies exist near *Teratorn*-like virus	Unidentified (small number of blast hits)	
*E. lucius*	partially degraded	~ 8	integrated (contig-mediated link between *Teratorn*-like virus and genomic region)	–	Unidentified (small number of blast hits)	
*P. magnuspinnatus*	intact	–	unkonwn	–	Unidentified (small number of blast hits)	
*P. argenteus*	unknown (contigs too short)	–	unknown	–	Unidentified (contigs too short)	
*O. latipes*	intact	~ 25, ~ 5	integrated (BAC sequencing)	Fused	TIRs of *piggyBac*-like element exist	2 subtypes
*A. limnaeus*	intact	1–2, ~ 4	likely integrated (scaffold-mediated link between *Teratorn*-like virus and genomic region)	some *piggyBac* copies exist near *Teratorn*-like virus	Unidentified (gaps between *Teratorn*-like virus and genomic region)	2 subtypes
*N. furzeri*	intact	1–2	unknown	Consistent link between *piggyBac* and *Teratorn*-like virus	Unidentified (small number of blast hits)	2 subtypes?
*K. marmoartus*	intact	–	unknown	–	Unidentified (contigs too short)	2 subtypes
*A. citrinellus*	intact	–	likely integrated (scaffold-mediated link between *Teratorn*-like virus and genomic region)	–	Unidentified (small number of blast hits)	
*O. niloticus*	partially degraded	~ 12, ~ 2	integrated (fosmid sequencing, scaffold-mediated link between *Teratorn*-like virus and genomic region, transposon insertion)	Consistent link between *piggyBac* and *Teratorn*-like virus sequence for subtype 1	Subtype 1: TIRs of *piggyBac*-like element exist Subtype 2: unidentified	2 subtypes
*N. brichardi*	intact	~ 2	likely integrated (scaffold-mediated link between *Teratorn*-like virus and genomic region)	–	Unidentified (small number of blast hits)	
*H. burtoni*	intact	~ 1	unknown	–	Unidentified (small number of blast hits)	
*P. nyererei*	unknown (contigs too short)	–	unknown	–	Unidentified (contigs too short)	at least 2 subtypes?
*M. auratus*	unknown (contigs too short)	–	unknown	–	Unidentified (contigs too short)	
*A. polyacanthus*	intact	–	likely integrated(scaffold-mediated link between *Teratorn* -like virus and genomic region)	–	Unidentified (small number of blast hits)	
*C. semilaevis*	degraded	–	integrated (ORF degradation)	some *piggyBac* copies exist near *Teratorn*-like virus	Unidentified (degradation of *Teratorn*-like virus sequences)	
*C. rhenanus*	unknown (contigs too short)	–	unknown	–	Unidentified (contigs too short)	
*L. crocea*	intact	~ 18	likely integrated (scaffold-mediated link between *Teratorn*-like virus and genomic region, transposon insertion)	Consistent link between *piggyBac* and *Teratorn*-like virus	Unidentified (gaps between *Teratorn*-like virus and genomic region)	
*M. albus*	intact (subtype2), degraded (subtype1)	~ 0.5, ~ 2	integrated (contig-mediated link between *Teratorn*-like virus and genomic region, ORF degradation)	Consistent link between *piggyBac* and *Teratorn-*like virus	Unidentified (small number of blast hits)	2 subtypes
*M. mola*	degraded	–	integrated (contig-mediated link between *Teratorn*-like virus and genomic region, ORF degradation)	Consistent link between *piggyBac* and *Teratorn*-like virus	Unidentified (small number of blast hits)	2 subtypes

In contrast, we did not get any significant hits against amphibian, chondrichthyes or sarcopterygi genomes, indicating that *Teratorn*-like viruses populate only the teleosts. Interestingly, tblastn search using sequences of another distantly-related alloherpesvirus species, Cyprinid herpesvirus 3 (CyHV-3), did not yield any positive hits other than *Teratorn*-like viruses, suggesting that *Teratorn*-like viruses are the only herpesvirus integrated in teleost genomes. Phylogenetic analysis based on amino acid sequences of five herpesvirus core genes demonstrated that *Teratorn*-like viruses are closely related to each other and form a cluster within *Alloherpesviridae* (Fig. 1b, Additional file 3: Figure S2). Indeed, nearly all *Teratorn*-like viruses showed high sequence similarity to medaka *Teratorn* (~ 70% nucleotide sequence identity), except for ones from *K. marmoratus* and *A. polyacanthus* (low nucleotides sequence identity). Although the relationship with other alloherpesviruses is unclear (i.e. sister group to *Ictalurivirus* or sister group to all other alloherpesviruses, Additional file 4: Figure S3), the fact that *Teratorn*-like viruses contain all 13 alloherpesvirus core genes [12] indicates that they belong to *Alloherpesviridae*.

The patchy distribution of *Teratorn*-like viruses among teleost fishes might result from multiple independent endogenization events in teleosts. Indeed, the phylogeny of *Teratorn*-like viruses exhibits little correlation with that of the hosts, except for those at the tip of some branches in cichlids and salmonids (Fig. 1a and b). In addition, for almost all pairs of species, the dN/dS ratio of herpesvirus genes is much lower than one (Additional file 5: Table S2), implying that viral genes are required for their transmission. Thus, *Teratorn*-like viruses may have been transmitted to each teleost lineage as the viral form. However, vertical inheritance of *Teratorn*-like viruses from a common ancestor of these teleost species cannot be ruled out.

**Fig. 2:**
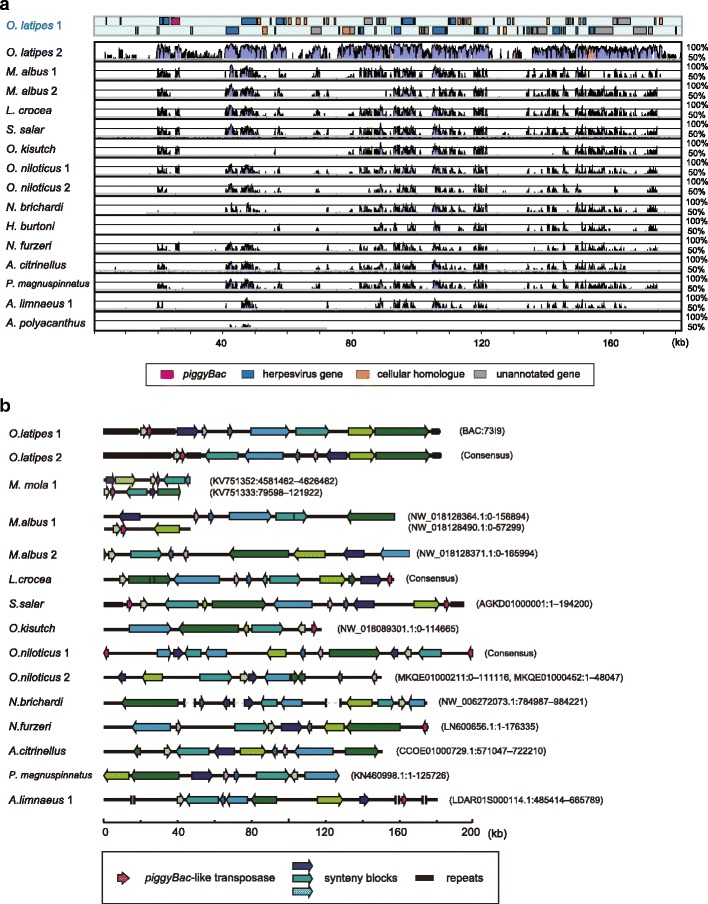
*Teratorn*-like viruses encode a series of herpesvirus genes **a** A homology plot of *Teratorn*-like virus sequences compared to medaka *Teratorn* subtype 1, visualized by VISTA. Blue and white regions indicate coding and non-coding regions, respectively. Colored boxes in the two bars above the histograms indicate the positions of forward- and reverse-oriented predicted genes of medaka *Teratorn* subtype 1 (magenta, *piggyBac*-like transposase gene; blue, herpesvirus-like genes; orange, cellular homologs; gray, unannotated genes). All sequences displayed in this plot are the same as those in (**b**). **b** Structures of *Teratorn*-like viruses in several teleost fish species. Conserved synteny blocks are depicted by arrows of the same color. Magenta arrows indicate the *piggyBac*-like transposase gene. Sources of each sequence are described on the right. These sequences were (1) extracted from contigs or scaffolds (*M. mola*, *M. albus*, *S. salar*, *O. kisutch*, *N. brichardi*, *N. furzeri*, *A. citrinellus*, *A. limnaeus* and *A. polyacanthus*), or (2) reconstructed by conjugating multiple contigs (subtype 2 *Teratorn* of *O. latipes*, *L. crocea* and *O. niloticus*) or BAC sequencing (subtype 1 *Teratorn* of *O. latipes*)

**Fig. 3:**
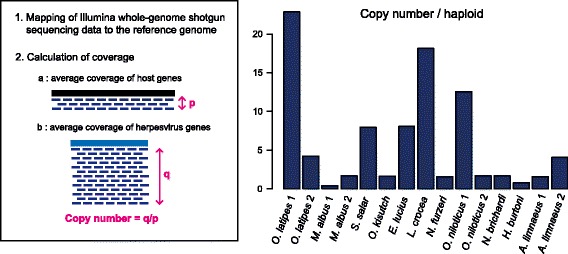
*Teratorn*-like viruses exist in multiple copies in host genomes Estimated copy number of *Teratorn*-like viruses in the genomes of some teleost fishes. Copy number was estimated by mapping of Illumina short read data against reference genome data, calculating the coverage at each nucleotide position, and then dividing the average coverage value of herpesvirus core genes by the average coverage of the coding region of host genes

For some species, we were able to obtain contigs or scaffolds over 100-kb in length that included *Teratorn*-like virus sequences (Fig. 2a, b). Gene annotation revealed that some of them contain a set of intact virus genes (Additional file 6: Text S1), suggesting that *Teratorn*-like viruses are maintained intact in those species. To examine the possibility of intragenomic propagation, we estimated the copy number of *Teratorn*-like viruses by mapping whole-genome shotgun reads of several teleost species to their reference genome (see Methods). We then calculated the putative copy number, by dividing the average coverage of herpesvirus core genes by that of all host genes, assuming that the copy number is proportional to the depth of coverage. We found that *Teratorn*-like viruses are present in multiple copies in some teleost species (Copy number per haploid genome: ~ 30 in medaka (*O. latipes*), ~ 18 copies in yellow croaker (*L. crocea*), ~ 14 copies in Nile tilapia (*O. niloticus*), ~ 8 copies in Atlantic salmon (*S. salar*) and northern pike (*E. lucius*), ~ 6 copies in annual killifish (*A. limnaeus*), 2–5 copies in Coho salmon (*O. kisutch*), 2–3 copies in swamp eel (*M. albus*); Fig. 3, Table 2, Additional file 7: Table S3). In addition, copy number varies for some pairs of closely related species (e.g. *S. salar* and *O. kisutch*, subtype 1 *O. niloticus*, *N. brichardi* and *H. burtoni*, see Fig. 1). Thus, *Teratorn*-like viruses may have increased their copy number within host genomes, as is the case in medaka [12]. Together, these data demonstrate that *Teratorn*-like viruses are teleost-specific, widely distributed in teleost genomes, and retain the capacity for propagation.

### *piggyBac*-like elements are frequently located close to or inside *Teratorn*-like viruses

We previously reported that *piggyBac*-herpesvirus fusion occurred in medaka (*O. latipes*), yellow croaker (*L. crocea*), Nile tilapia (*O. niloticus*), ocean sunfish (*M. mola*), and turquoise killifish (*N. furzeri*) [12]. In the present study, we identified four additional fish species that contain *piggyBac*-like transposons close to or inside *Teratorn*-like viruses (annual killifish (*A. limnaeus*), Atlantic salmon (*S. salar*), Coho salmon (*O. kisutch*) and Asian swamp eel (*M. albus*) (Fig. 2a, b, magenta)), suggesting that fusion with a *piggyBac*-like transposon may be a general phenomenon for the herpesvirus genus. However the location of the *piggyBac*-like transposase gene inside *Teratorn*-like virus is not necessarily conserved among these fishes. Although they tend to be located at the edges of *Teratorn*-like viruses (e.g. salmonids, yellow croaker, killifish, Nile tilapia), various herpesvirus genes were found to be next to the transposase (Fig. 2b, Additional file 6: Text S1). This suggests that fusion occurred on multiple occasions. By contrast, in swamp eel, ocean sunfish and medaka, the *piggyBac*-like transposase gene is located within the herpesvirus-like sequences, and its neighboring genes are always ORF60 and ORF54, which are respectively the second and most proximal (Fig. 2b), suggesting that this fusion preceded the invasion into those species. Taken together, these data demonstrate that the fusion of *piggyBac*-like elements and *Teratorn*-like viruses occurred frequently in teleosts.

### Co-existence of *piggyBac*-like elements and *Teratorn*-like viruses

**Fig. 4:**
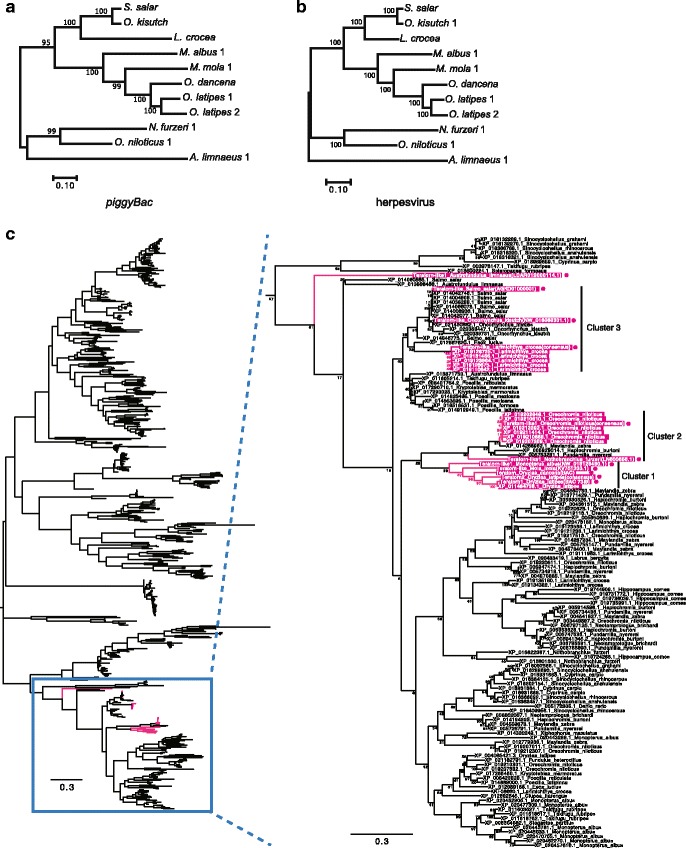
Phylogenetic comparison between *piggyBac*-like elements and herpesvirus-like genes (**a**) A Maximum-likelihood tree based on the nucleotide sequences of *piggyBac*-like transposase genes inside *Teratorn*-like viruses is shown. General time reversible model was used as the substitution model, and evolutionary rate differences among sites was modeled by discrete gamma distribution. A total of 1518 positions were used in the final dataset. **b** A maximum-likelihood tree based on the concatenated nucleotide sequences of five herpesvirus genes (DNA polymerase, DNA helicase, terminase, major capsid protein, membrane glycoprotein) is shown. General time reversible model was used as the substitution model, and evolutionary rate differences among sites was modeled by discrete gamma distribution. A total of 13,683 positions were used in the final dataset. **c** A maximum-likelihood tree based on the amino acid sequences of all annotated *piggyBac*-like transposase genes in teleosts is shown. Expansion of the clade surrounded by the blue square is shown on the right. Magenta indicates *piggyBac*-like transposase genes inside *Teratorn*-like viruses. JTT model was used as substitution model, and evolutionary rate differences among sites were not modeled. The bar represents the number of substitutions pre site. A total of 324 positions were used in the final dataset. Note the existence of three clusters containing *piggyBac*-like elements fused with *Teratorn*-like viruses, the phylogeny of which is highly correlated with that of *Teratorn*-like viruses (see Fig. 1b). Sequences used for the phylogenetic tree in (a) are marked by magenta circles. The scale bars represent the number of substitutions per site

The discovery that *piggyBac*-like elements and *Teratorn*-like viruses are frequently fused led us to hypothesize an evolutionary association between the two elements. Indeed, we have yet to find any other DNA transposons inside *Teratorn*-like viruses, unless viral sequences have been degraded. To test our hypothesis, we compared the phylogeny of *piggyBac*-like transposase genes and herpesvirus-like genes. We first selected *Teratorn*-like viruses fused with *piggyBac*-like elements and found that the topology of the two phylogenetic trees was nearly identical (Fig. 4a, b). In addition, the level of synonymous divergence (dS) between the two was comparable (Additional file 8: Table S4), implying co-evolution of the two mobile elements. Second, we examined whether a specific group of *piggyBac*-like elements tend to fuse with *Teratorn*-like viruses. We collected amino acid sequences of all annotated *piggyBac*-like transposase genes found in teleosts from a non-redundant protein database (PSI-Blast; five times iterations; e-value cutoff, 1e-50; query, transposase sequence of subtype 1 medaka *Teratorn*) and performed a phylogenetic analysis. We found that, of all *piggyBac*-like elements, *piggyBac*-like elements fused with *Teratorn*-like viruses are closely related to each other, but not monophyletic (Magenta in Fig. 4c, Additional file 9: Figure S4). Despite low bootstrap values, and inconsistent results produced by maximum-likelihood analysis and neighbor-joining analysis, we nonetheless show that there are at least three clusters containing *piggyBac*-like elements fused with *Teratorn*-like viruses (Fig. 4c, Additional file 9: Figure S4). Cluster 1 (medaka (*O. latipes*), ocean sunfish (*M. mola*) and swamp eel (*M. albus*)) consists of only *piggyBac*-like elements fused with *Teratorn*-like viruses, further supporting the idea that the fusion occurred prior to their invasion into these species (see above). By contrast, cluster 2 (cichlids and turquoise killifish (*N. furzeri*)) and cluster 3 (salmonids and yellow croaker (*L. crocea*)) include *piggyBac*-like elements which exist alone (i.e. no *Teratorn*-like viruses were found in their vicinity). However, all fish species included in both clusters have *Teratorn*-like viruses in their genomes, either in the fused form or independently, and the topology of *piggyBac*-like elements is highly correlated with that of *Teratorn*-like viruses (Compare Fig. 1b and Fig. 4c). Thus, *piggyBac*-like elements and *Teratorn*-like viruses in the two clusters may also have a specific relationship with each other. Together, these data suggest co-evolution of the *piggyBac*-like elements and *Teratorn*-like viruses, which could facilitate the fusion of these two mobile genetic elements in host genomes.

## Discussion

Our comprehensive genomic survey demonstrated that *Teratorn*-like viruses are present in a wide range of teleost fishes. Although we cannot exclude the possibility that positive hits in a genomic database search can be a result of contamination of draft genomes with exogenous virus DNA, the following facts provide support for the existence of at least some genuine genomic integrants. First, we confirmed the link between virus-like sequences and other endogenous genomic regions for 19 of the 29 identified elements, indicating insertions (connection in contigs, eight elements; connection in scaffolds, nine elements; connection in BAC or fosmid clones, two elements). Second, some virus-like sequences are interrupted by various types of transposons (three of the 29 elements). Finally, we observed ORF degradation in *Teratorn*-like virus sequences, which is unlikely in exogenous viruses (four of the 29 elements, Table 2).

*Teratorn*-like viruses identified in this study are phylogenetically close to each other, forming a cluster inside *Alloherpesviridae*. To date, four genera have been established in *Alloherpesviridae*; *Batrachovirus*, *Cyprinivirus*, *Ictalurivirus* and *Salmonivirus* [14]. However, given the evolutionary distance from these genera and broad distribution in teleosts, we propose that this group of *Teratorn*-like viruses should be regarded as a separate genus.

Recently (in fact, during the preparation of this manuscript), Asward and Katzourakis reported the *Teratorn*-like viruses as a sister group to *Alloherpesviridae* [13] based on the phylogenetic analysis of DNA polymerase gene, while our analysis located it inside *Alloherpesviridae*. At this level, the result could change depending on the number of elements analyzed (25 for the present study, and 15 for that of Asward and Katzourakis [13]). Nonetheless, the fact that *Teratorn*-like viruses contain all 13 core genes conserved among alloherpesviruses suggests that they belong to *Alloherpesviridae* [12].

*Teratorn*-like viruses as a whole appear to be unique in having a high tendency for endogenization. Our database search consistently failed to identify endogenous herpesviruses in alloherpesvirus species other than *Teratorn*-like viruses. We further found that *Teratorn*-like viruses, sometimes present in multiple copies, are frequently located near *piggyBac*-like elements (at least 12 of the 29 viral elements, Figs. 1, 2). Thus, acquisition of the *piggyBac*-like elements could be a major driving force for integration and propagation in many teleost species. Indeed, our previous analysis focusing on medaka species revealed the complete fusion of *Teratorn*-like viruses and the *piggyBac* transposon (i.e. transposase gene and herpesvirus-like genes are flanked by terminal inverted repeats (TIRs)); the fused form retaining the ability to transpose [12]. In addition, terminal sequences of *Teratorn*-like viruses are often bordered by TIRs of *piggyBac*-like elements in Nile tilapia [12] and Atlantic salmon [13], further supporting the fusion of the two elements in these two fish species. However, we could not identify the exact integration sites of the *Teratorn*-like virus for other fish species, probably due to gaps, short contigs and low copy number in the genome assembly data (Table 2). Of course, not all *Teratorn*-like viruses have *piggyBac*-like elements in their vicinity. This could simply be due to an incomplete assembly of genome data. Alternatively, these viruses may have invaded host genomes accidentally or by an unknown mechanism.

**Fig. 5:**
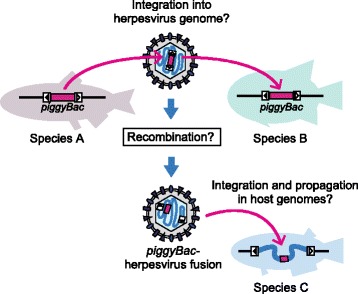
Model: *piggyBac*-herpesvirus fusion resulting from virus-mediated transfer of transposons Fusion of *piggyBac*-like elements and *Teratorn*-like viruses could have originated from virus-mediated transfer of transposons across teleost fish species. Since herpesviruses invade the nucleus of host cells to establish latent infection, transfer of transposons between the host genome and virus DNA is theoretically possible. In some cases, rearrangement might occur within the virus genome to form a genuine *piggyBac*-herpesvirus fusion, making it possible for the virus to integrate and propagate in the host genome

One of the aims of this study was to clarify the evolutionary relationship between *piggyBac*-like elements and *Teratorn*-like viruses. Intriguingly, phylogenetic trees for the two mobile elements, in cases when they are present next to each other in the genome, are similar, although not monophyletic, for the *piggyBac*-like elements. Although simple coincidence (i.e. independent accidental fusion in each fish lineage) remains a possibility, co-evolution is a plausible explanation for this phylogenetic result. Indeed, both the fused and the separate forms of these two elements tend to co-occur in teleost genomes (Fig. 4c), suggesting a long-term intimate relationship between them. A possible explanation for this association is that *Teratorn*-like viruses utilize *piggyBac* transposase for integration into the host chromosome and intragenomic propagation, while *piggyBac* elements propagate across hosts and species with the aid of *Teratorn*-like viruses (Fig. 5). It is thought that transposable elements are transferred horizontally across species via viruses [15–19] or other parasites [18–21]. Our data is in agreement with the idea of virus-mediated transmission of transposons across species. It will be important to isolate virions of *Teratorn*-like viruses that contain *piggyBac*-like elements in order to gain direct evidence for this idea.

The frequent fusion between *piggyBac*-like elements and *Teratorn*-like viruses raises another question of why only this combination was observed. For *Teratorn*-like viruses, *piggyBac*-like transposons are suitable in that they can carry a large cargo of DNA fragments, up to around 100 kb, the size comparable with that of herpesvirus [22, 23]. However, we do not know why *piggyBac*-like transposons specifically carry *Teratorn*-like viruses as cargo. *Teratorn*-like viruses may have some biological and structural features that attract or benefit *piggyBac*-like transposons. More detailed characterization of *Teratorn*-like viruses, including study of the life cycle, will be needed to answer these questions.

*Teratorn*-like viruses, after fusion with *piggyBac*-like elements, affect host organisms as EVEs, in a manner different from exogenous counterparts. Indeed, EVEs can behave as genomic parasites, akin to transposable elements [1, 24, 25], and/or as symbionts (e.g. offer immunity to exogenous viruses, construction of novel gene regulatory networks, co-option for specific host functions) [1, 26–30]. Further functional analysis of *Teratorn*-like viruses may uncover additional biological properties of herpesviruses.

## Conclusions

Our comprehensive genomic survey reveals that the novel herpesvirus-like sequences (named *Teratorn*-like viruses) are widely distributed in teleost genomes, and are frequently fused with *piggyBac*-like transposable elements. Phylogenetic analysis suggests long-term co-evolution of *piggyBac*-like elements and *Teratorn*-like viruses, which could have facilitated their fusion for intragenomic propagation. Our study provides unique examples of intragenomic propagation of herpesviruses in teleost genomes, with the aid of *piggyBac*-like transposons.

## Methods

### Search for *Teratorn*–like viruses in teleost fish species

A tblastn search of 15 herpesvirus genes of *Teratorn* (DNA polymerase, DNA helicase, primase, ATPase subunit of terminase, major capsid protein, membrane glycoprotein, capsid triplex protein, capsid maturation protease, ORF34, ORF37, ORF44, ORF54, ORF56, ORF60, ORF64) was carried out against all available teleost genomes using default parameters. In addition, tblastn of four genes (DNA polymerase, DNA helicase, DNA packaging terminase and major capsid protein) was performed against amphibians, chondrichthyes or sarcopterygi in the NCBI blast web browser. Contigs or scaffolds that include a series of herpesvirus-like sequences were screened as follows. First, locations of the 15 herpesvirus genes were identified by tblastn. After merging the genomic loci, which are within 60 kb of one another, sequences of the defined region and the flanking 40 kb region were extracted from the draft genomes using BEDtools [31]. The list of teleost fish species used in the genomic search of *Teratorn*-like viruses are in Additional file 10: Table S5.

### Gene annotation of *Teratorn*-like viruses

Gene annotation was initially carried out using the GeneMarkS web server with “Virus” selected as the sequence type [32]. If adjacent multiple ORFs seemed to be derived from a single gene (i.e. different portions of the same gene were obtained as blastp output), gene annotation by GENSCAN web server [33] was used to generate a more plausible gene model including introns.

### *Teratorn*-like virus sequence comparisons

Homology plot of *Teratorn*-like viruses against subtype 1 medaka *Teratorn* was created with VISTA program with the Shuffle-LAGAN option (http://genome.lbl.gov/vista/index.shtml). To compare the structures of *Teratorn*-like viruses, a dot plot matrix against subtype 1 medaka *Teratorn* was created using the mafft online server (http://mafft.cbrc.jp/alignment/software/).

### Copy number estimation of *Teratorn*-like viruses

Reference genome data were reconstructed as follows. First, *Teratorn* or *Teratorn*-like virus sequences were masked from the genome by blastn, followed by maskFastaFromBed command of BEDtools [31]. Then, the masked genome data were conjugated with *Teratorn*-like virus sequences. Illumina whole-genome shotgun read data were downloaded from DDBJ Sequence Read Archives (accession numbers are listed in Additional file 7: Table S3). After filtering out low-quality reads by trimmomatic v0.33 [34], reads were aligned to the reconstructed reference genome using BWA(Burrows-Wheeler Aligner)-MEM [35], using default parameter settings. After converting the output sam files into bam files, coverage at each position in all coding regions was counted by the coverageBed command of BEDtools with the –d option. Copy numbers of *Teratorn*-like sequences were then calculated by dividing the average coverage of 15 herpesvirus genes (DNA polymerase, DNA helicase, primase, ATPase subunit of terminase, major capsid protein, membrane glycoprotein, capsid triplex protein, capsid maturation protease, ORF34, ORF37, ORF44, ORF54, ORF56, ORF60, ORF64) by the average coverage of the remainder of the host genes (all species except for *N. furzeri*) or the partial region of 19 host genes (*N. furzeri*), assuming that copy number is proportional to the depth of read coverage.

### Phylogenetic analysis

For the phylogenetic analysis of *Teratorn*-like viruses, nucleotide sequences of six herpesvirus-like genes (DNA polymerase, DNA helicase, major capsid protein, capsid triplex protein, DNA packaging terminase and membrane glycoprotein) were obtained by tblastn search against teleost genomes as described above, followed by extraction of sequences by BEDtools. For species in which each herpesvirus-like gene was separated into multiple small contigs, the gene sequence was artificially reconstructed by manually conjugating the small contigs. Next, nucleotide alignments were constructed using MUSCLE in MEGA7 [36] or MAFFT [37], followed by removal of poorly aligned regions using trimAl with either a –strict or –strictplus options and/or using the manual procedure. Preliminary neighbor-joining trees were then constructed for each gene using MEGA7 with 1000 bootstraps (Kimura 2-parameter model, uniform evolutionary rates among sites). For species that contained multiple blast hits for a single gene, we regarded sets of sequences that had pairwise nucleotide sequence identity of more than 90% as a single subtype and chose a single copy for further phylogenetic analysis (see Additional file 1: Figure S1).

Amino acid sequences of herpesvirus genes were obtained by converting the nucleotide sequences to amino acid sequences (*Teratorn*-like viruses) or by searching for them in GenBank (other herpesvirus species). Multiple alignments were constructed using MUSCLE in MEGA7 (DNA polymerase, DNA helicase, major capsid protein and DNA packaging terminase) or PROMALS3D [38] (capsid triplex protein and membrane glycoprotein), followed by removal of poorly aligned regions using trimAl with the –strict option. Maximum-likelihood trees were constructed for five herpesvirus genes (DNA polymerase, DNA helicase, major capsid protein, DNA helicase and capsid triplex protein), as well as concatenation of the same genes, using MEGA7 with 100 bootstraps (Le Gascuel 2008 model, discrete gamma distribution with five rate categories, Fig. 1b, Figure S3 and S4).

To characterize the host phylogeny, nucleotide sequences of 17 host genes (*enc1*, *ficd*, *glyt*, *gpr85*, *kiaa1239*, *myh6*, *panx2*, *plagl2*, *ptchd1*, *rag1*, *rag2*, *rh*, *ripk4*, *sh3px3*, *tbr*, *vcpip*, *zic1* [39]) were extracted from teleost genomes and the coelacanth genome (*Latimeria chalumnae*), using tblastn and BEDtools as described above. Codon alignments were constructed by MUSCLE in MEGA7, followed by manual trimming of poorly aligned regions. A Bayesian inference tree was constructed from the concatenation of the 17 genes using MrBayes3.2 [40] (General time reversible model, discrete gamma distribution with four rate categories, Fig. 1a). Four individual runs of MCMC were performed with four chains for 1,000,000 generations, with trees being sampled every 200 generations. The initial 25% of trees were discarded and the rest were used for the construction of the consensus tree.

To compare the phylogenies of herpesvirus-like genes and the *piggyBac*-like transposase gene inside *Teratorn*-like viruses, maximum-likelihood analysis was performed for each element using MEGA7 with 200 bootstraps (general time reversible model, discrete gamma distribution with five rate categories, Fig. 4a, b). For herpesvirus-like genes, concatenated nucleotide sequences of DNA polymerase, DNA helicase, major capsid protein, membrane glycoprotein and terminase were used. For the *piggyBac*-like element, a single copy was selected per each species or subtype (see Additional file 9: Figure S4).

To characterize the phylogeny of *piggyBac*-like elements in teleosts, amino acid sequences of the transposase genes were obtained by PSI-Blast search against a non-redundant protein database of all teleosts (five times iterations; e-value cutoff, 1e-50; query, transposase sequence of subtype 1 medaka *Teratorn*). A multiple alignment was constructed using mafft [37] with default parameters, followed by trimming of poorly aligned regions using trimAl with the –strict option. Sequences with greater than 160 amino acids were selected for phylogenetic analysis. Maximum-likelihood and neighbor-joining analyses were performed using MEGA7 with 200 bootstraps (Jones-Taylor-Thornton model, uniform evolutionary rates among sites, Fig. 4a, b).

Gene(s), species, and parameters utilized for all phylogenetic analyses in this study are summarized in Additional file 11: Table S6.

### Calculation of pairwise sequence divergence

Multiple codon alignments of herpesvirus-like genes, *piggyBac*-like transposase genes and host genes were built up as described above. Pairwise synonymous and non-synonymous sequence divergences were calculated by the modified Nei-Gojobori model (assumed transition/transversion bias = 2), using MEGA7 [37].

## Additional files

Additional file 1: **Figure S1.** Phylogenetic trees of all *Teratorn*-like virus copies obtained by blast search Neighbor-joining trees of each herpesvirus gene are shown. Kimura’s two-parameter model, assuming uniform evolutionary rates among sites, was used as nucleotide substitution model. For DNA polymerase, major capsid protein and membrane glycoprotein, phylogenetic trees were also constructed from the first and second half of the genes, since some sequences contain only a part of the coding region. For terminase, phylogenetic trees were independently constructed for each of the three exons. Numbers above the trees indicate the corresponding regions relative to the CDS of subtype 1 medaka *Teratorn*. Sequences marked by magenta were used for phylogenetic trees in Fig. 1b and Fig. 4b, those marked in orange were used for phylogenetic trees in Fig. S2 and S3, and those marked with cyan are same as those in Fig. 2. The bars represent the number of substitutions per site. (PDF 234 kb)

Additional file 2: **Table S1.** Result of tblastn search against teleost genomes (XLSX 223 kb)

Additional file 3: **Figure S2.** Phylogenetic trees of each herpesvirus gene Maximum-likelihood trees of each herpesvirus gene are shown. Le and Gascuel’s model (2008), considering evolutionary rate differences among sites by discrete gamma distribution, was used as protein substitution model. *Teratorn*-like viruses are depicted in blue. The bars represent the number of substitutions per site. (PDF 143 kb)

Additional file 4: **Figure S3.** Evolutionary relationships of *Teratorn*–like viruses with other herpesviruses Maximum-likelihood trees of DNA packaging terminase gene, the only gene confidently conserved among *Herpesvirales*, are shown. All identified *Teratorn*-like viruses (**a**) or part of elements (**b**) used for phylogenetic analysis. Le and Gascuel’s model (2008), considering evolutionary rate differences among sites by discrete gamma distribution, was used for protein substitution. Species belonging to *Caudovirales* (bacteriophage), *Herpesviridae*, *Malacoherpesviridae*, *Alloherpesviridae* and *Teratorn*-like viruses are depicted by green, orange, purple, dark blue and light blue, respectively. Note that relationships with alloherpesviruses are different between the two analyses, presumably due to the difference in the number of sequences. The bars represent the number of substitutions per site. (PDF 138 kb)

Additional file 5: **Table S2.** Pairwise synonymous and non-synonymous distance of herpesvirus genes and host genes among teleost fish species (XLSX 86 kb)

Additional file 6: **Text S1.** Sequences of representative *Teratorn*-like viruses identified in this study (TXT 2804 kb)

Additional file 7: **Table S3.** Copy number of *Teratorn*-like viruses estimated by whole-genome shotgun sequencing data (XLSX 76 kb)

Additional file 8: **Table S4.** Pairwise synonymous and non-synonymous distance of *piggyBac*-like transposase and herpesvirus genes (XLSX 71 kb)

Additional file 9: **Figure S4.** Neighbor-joining analysis of all *piggyBac*-like elements in teleosts Neighbor-joining tree based on the amino acid sequences of all annotated *piggyBac*-like transposase genes in teleosts is shown. Expansion of the clade surrounded by the blue square is shown on the right. Magenta indicates *piggyBac*-like transposase genes inside *Teratorn*-like viruses. JTT model was used as substitution model. Evolutionary rate differences among sites was not modeled. The bar represents the number of substitutions per site. A total of 324 positions were used in the final dataset. (PDF 166 kb)

Additional file 10: **Table S5.** Accession numbers of genome sequence data of teleost fish species (XLSX 47 kb)

Additional file 11: **Table S6.** Parameters used for phylogenetic analyses (XLSX 34 kb)

Additional file 12: **Text S2.** Trimmed alignments used for phylogenetic tree construction (TXT 3590 kb)

## Abbreviations

BAC: Bacterial artificial chromosome
Blast: Basic local alignment search tool
CDS: Coding DNA sequence
CyHV-3: Cyprinid herpesvirus 3
dN: Rate of non-synonymous divergence
dS: Rate of synonymous divergence
EVE: Endogenous viral element
HHV-6: Human herpesvirus 6
kb: Kilo base pair
ORF: Open reading frame
PSI-Blast: Position-specific iterated blast
TIR: Terminal inverted repeat
