# Supplementary material for: Fusion of piggyBac-like transposons and herpesviruses occurs frequently in teleosts
Source: Zoological Lett. 2018 Feb 21;4:6. doi: 10.1186/s40851-018-0089-8 (PMC5822658; doi:10.1186/s40851-018-0089-8)
Supplement: Supplementary file 1 — Figure S1. Phylogenetic trees of all Teratorn-like virus copies obtained by blast search Neighbor-joining trees of each herpesvirus gene are shown. Kimura’s two-parameter model, assuming uniform evolutionary rates among sites, was used as nucleotide substitution model. For DNA polymerase, major capsid protein and membrane glycoprotein, phylogenetic trees were also constructed from the first and second half of the genes, since some sequences contain only a part of the coding region. For terminase, phylogenetic trees were independently constructed for each of the three exons. Numbers above the trees indicate the corresponding regions relative to the CDS of subtype 1 medaka Teratorn. Sequences marked by magenta were used for phylogenetic trees in Fig. 1b and Fig. 4b, those marked in orange were used for phylogenetic trees in Fig. S2 and S3, and those marked with cyan are same as those in Fig. 2. The bars represent the number of substitutions per site. (PDF 234 kb) [file 40851_2018_89_MOESM1_ESM.pdf]

DNA polymerase

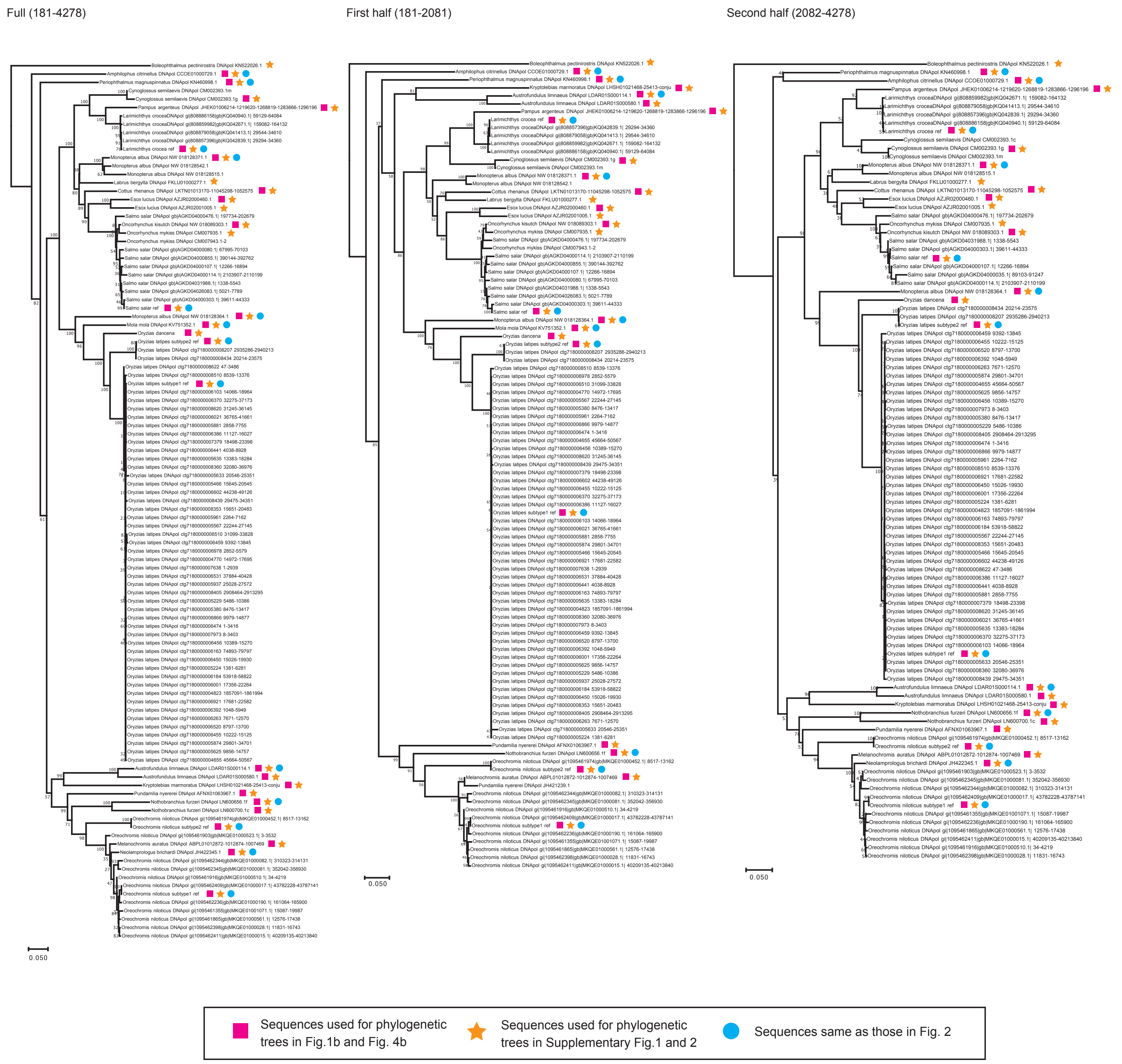

Figure S1

DNA helicase

Full (116-1758)

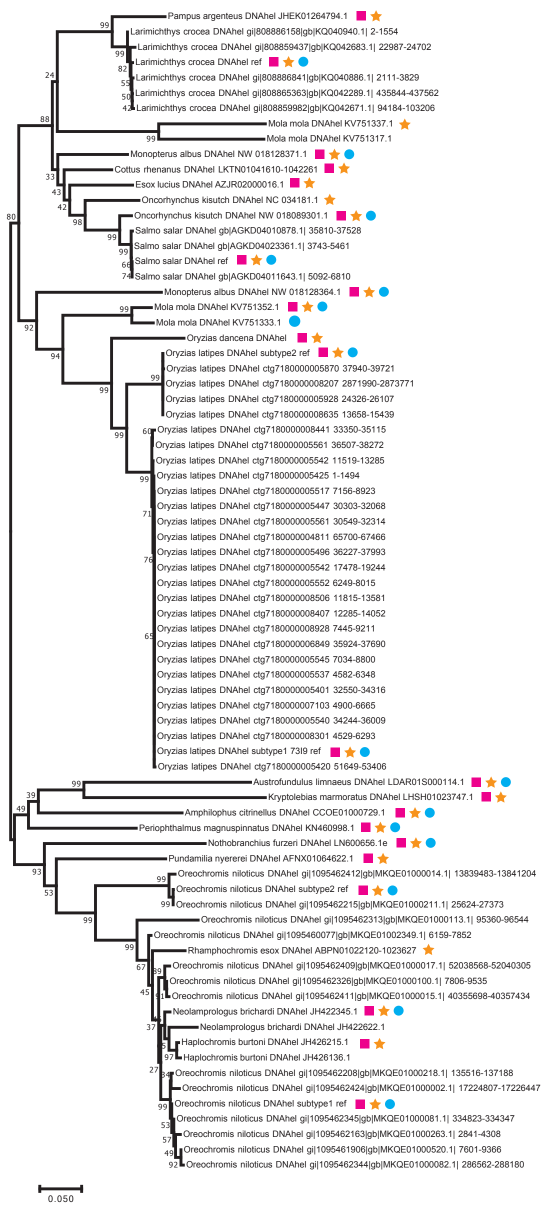

Capsid triplex protein

Full (2-863)

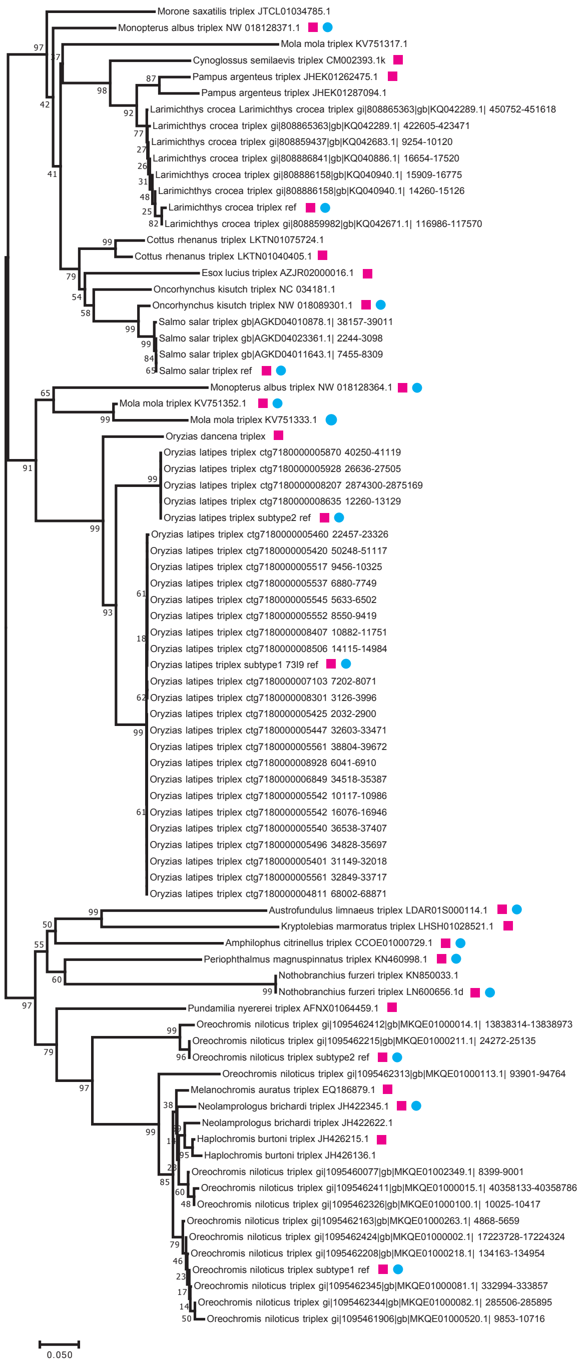

■ Sequences used for phylogenetic trees in Fig.1b and Fig. 4b ★ Sequences used for phylogenetic trees in Supplementary Fig.1 and 2 ● Sequences same as those in Fig. 2

Figure S1 (continued)

Major capsid protein

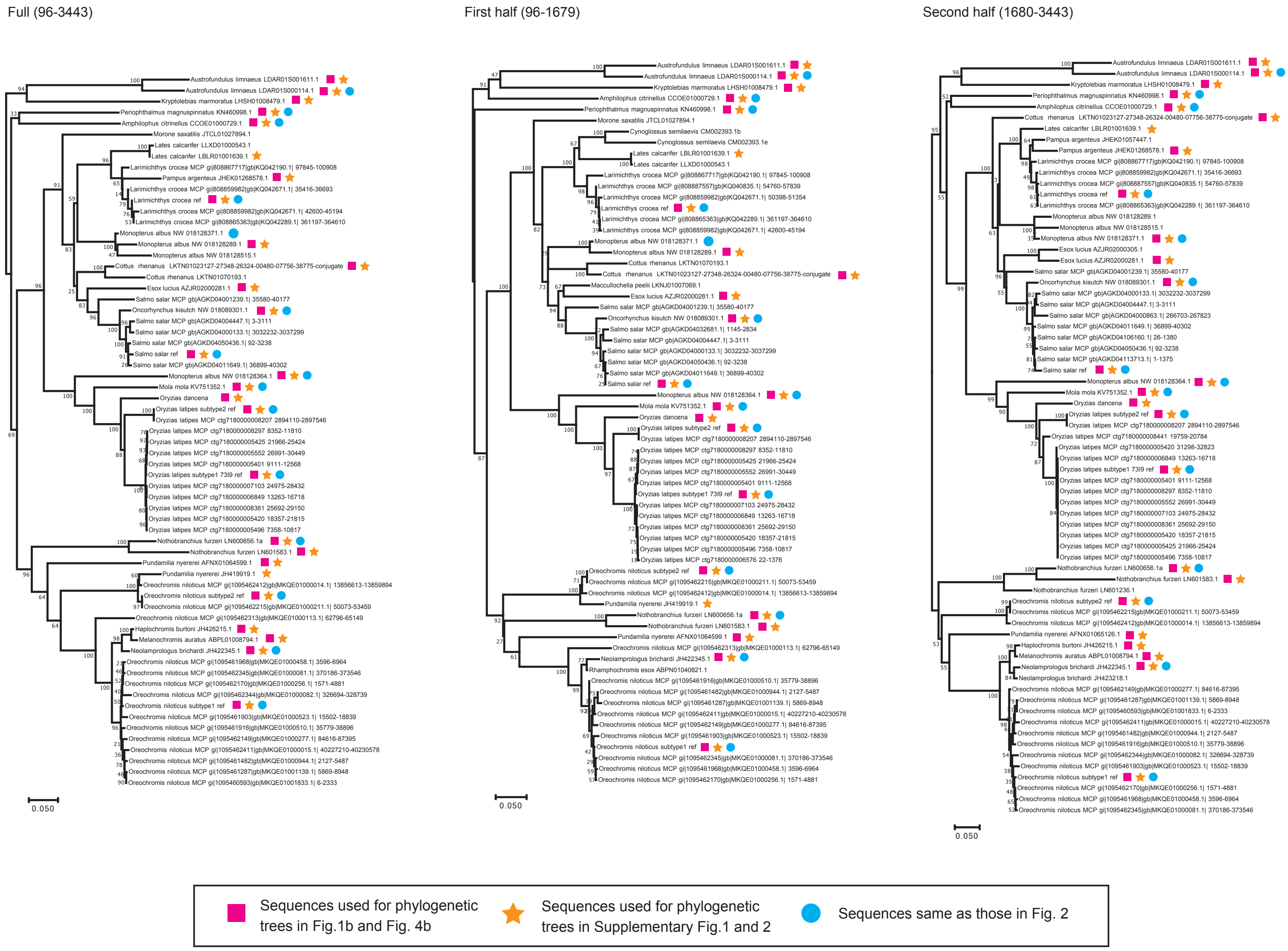

Figure S1 (continued)

Membrane glycoprotein

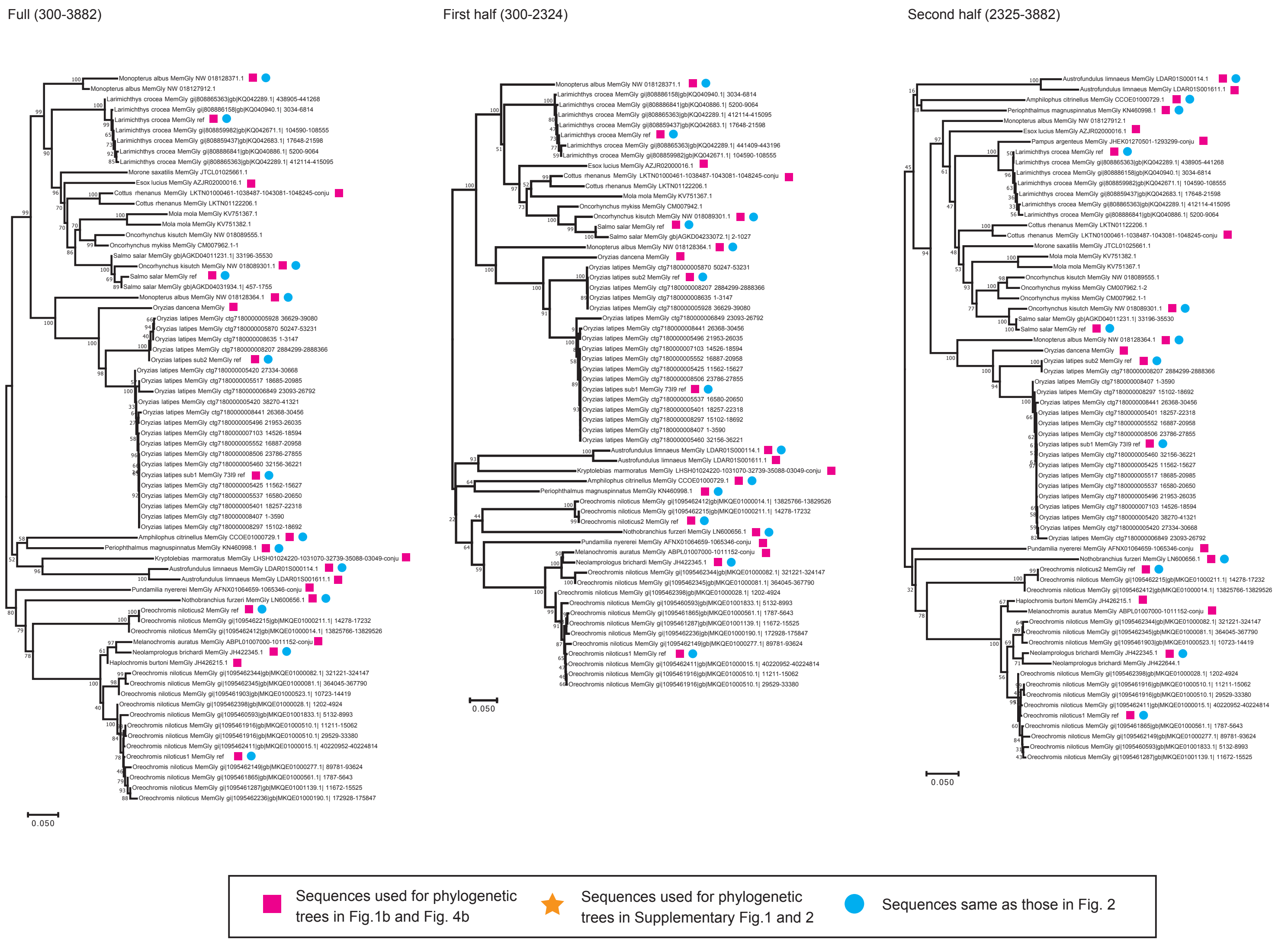

Figure S1 (continued)

DNA packaging terminase

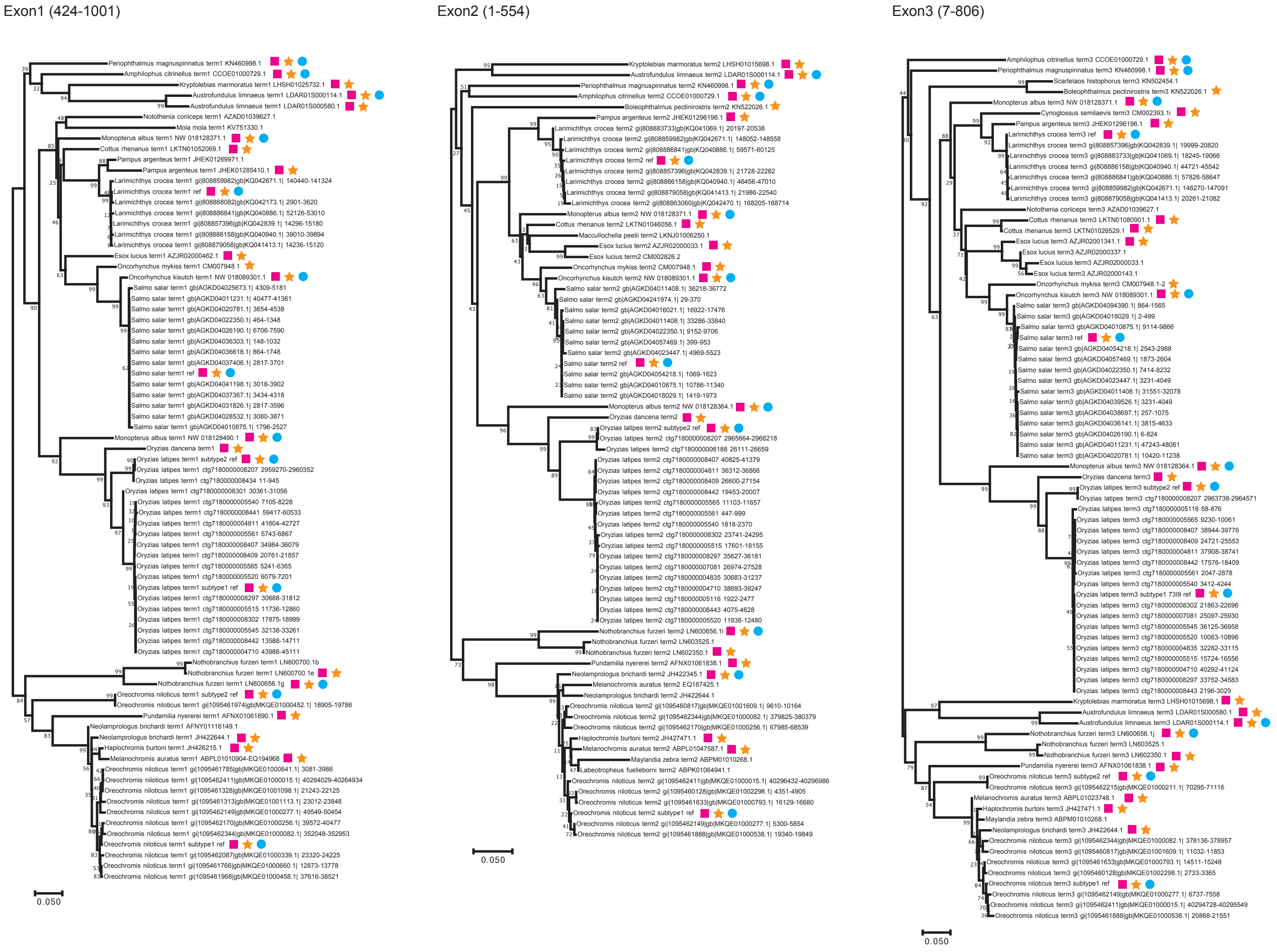

■ Sequences used for phylogenetic trees in Fig.1b and Fig. 4b

★ Sequences used for phylogenetic trees in Supplementary Fig.1 and 2

● Sequences same as those in Fig. 2

Figure S1 (continued)
