# Supplementary figures and images for: Fusion of piggyBac-like transposons and herpesviruses occurs frequently in teleosts
Source: Zoological Lett. 2018 Feb 21;4:6. doi: 10.1186/s40851-018-0089-8 (PMC5822658; doi:10.1186/s40851-018-0089-8)

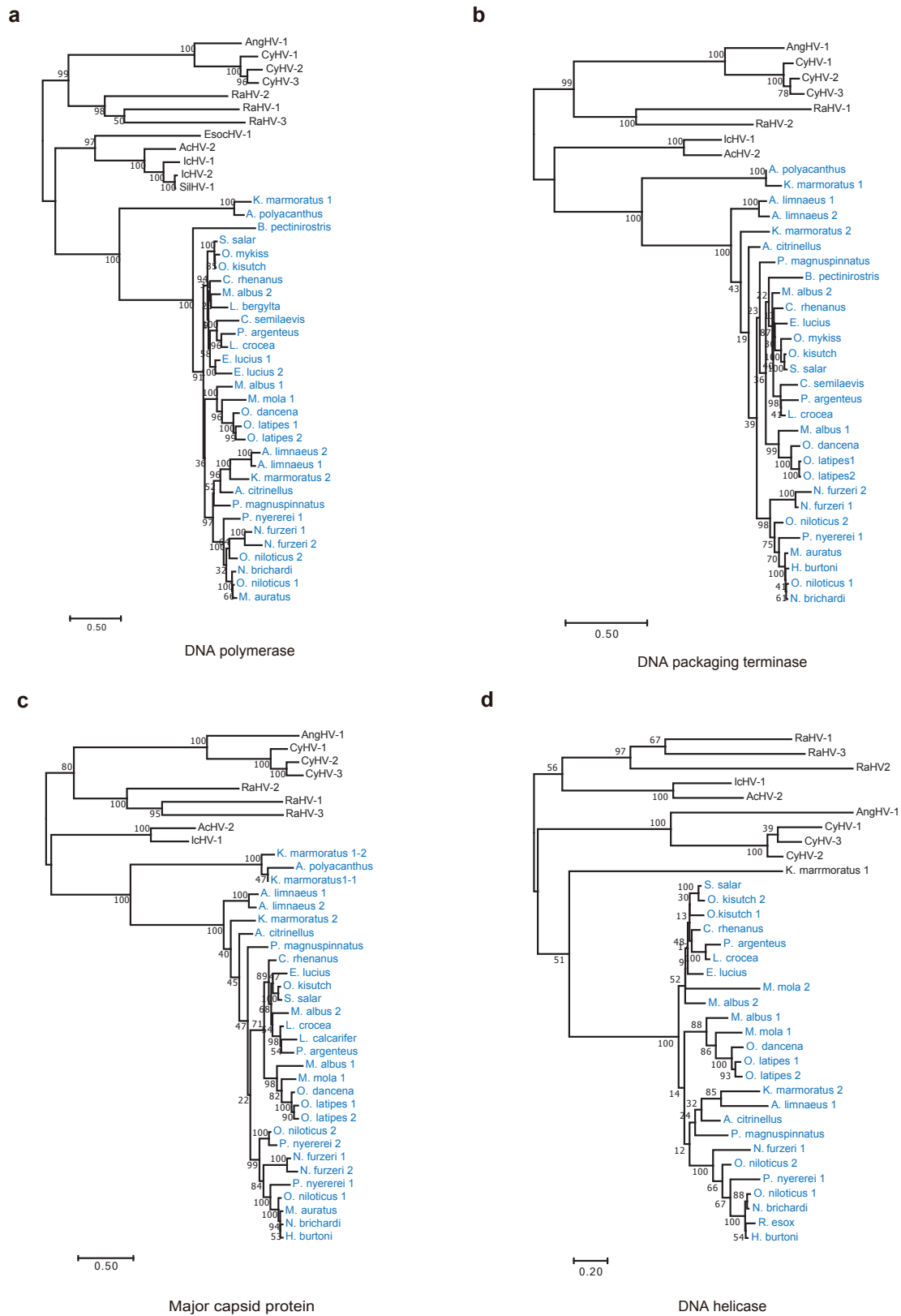

**Figure S2**

Supplement: Supplementary file 3 — Figure S2. Phylogenetic trees of each herpesvirus gene Maximum-likelihood trees of each herpesvirus gene are shown. Le and Gascuel’s model (2008), considering evolutionary rate differences among sites by discrete gamma distribution, was used as protein substitution model. Teratorn-like viruses are depicted in blue. The bars represent the number of substitutions per site. (PDF 143 kb) [file 40851_2018_89_MOESM3_ESM.pdf]

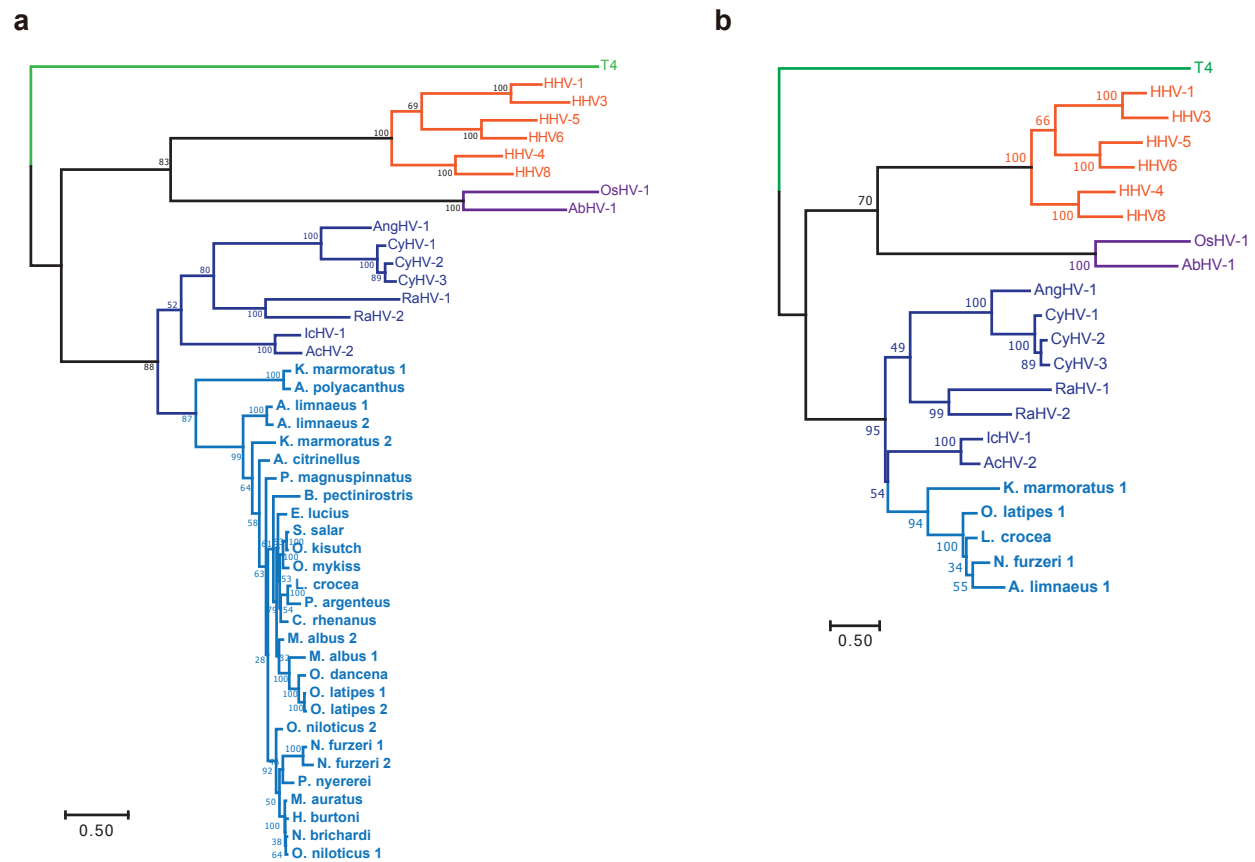

**Figure S3**

Supplement: Supplementary file 4 — Figure S3. Evolutionary relationships of Teratorn–like viruses with other herpesviruses Maximum-likelihood trees of DNA packaging terminase gene, the only gene confidently conserved among Herpesvirales, are shown. All identified Teratorn-like viruses (a) or part of elements (b) used for phylogenetic analysis. Le and Gascuel’s model (2008), considering evolutionary rate differences among sites by discrete gamma distribution, was used for protein substitution. Species belonging to Caudovirales (bacteriophage), Herpesviridae, Malacoherpesviridae, Alloherpesviridae and Teratorn-like viruses are depicted by green, orange, purple, dark blue and light blue, respectively. Note that relationships with alloherpesviruses are different between the two analyses, presumably due to the difference in the number of sequences. The bars represent the number of substitutions per site. (PDF 138 kb) [file 40851_2018_89_MOESM4_ESM.pdf]

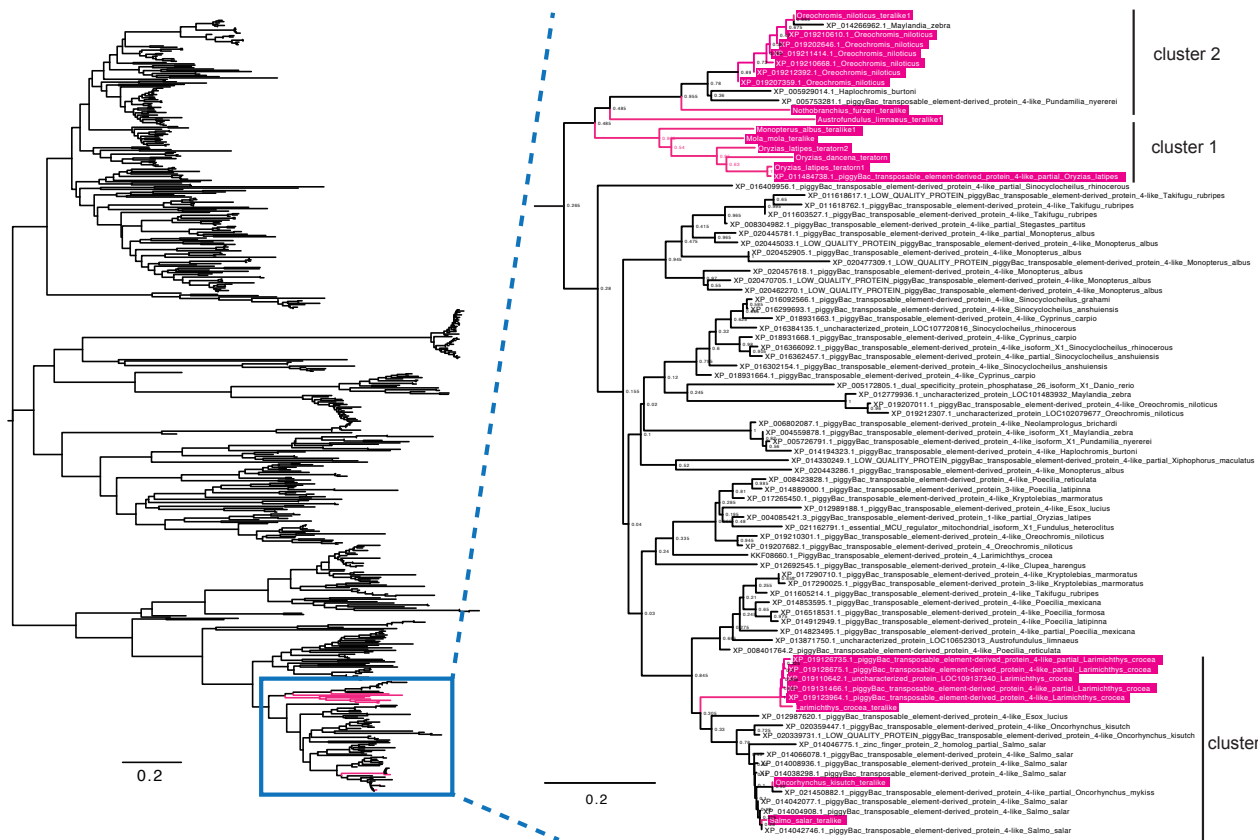

Figure S4

Supplement: Supplementary file 9 — Figure S4. Neighbor-joining analysis of all piggyBac-like elements in teleosts Neighbor-joining tree based on the amino acid sequences of all annotated piggyBac-like transposase genes in teleosts is shown. Expansion of the clade surrounded by the blue square is shown on the right. Magenta indicates piggyBac-like transposase genes inside Teratorn-like viruses. JTT model was used as substitution model. Evolutionary rate differences among sites was not modeled. The bar represents the number of substitutions per site. A total of 324 positions were used in the final dataset. (PDF 166 kb) [file 40851_2018_89_MOESM9_ESM.pdf]
